# RE: Water pipe (shisha) smoking among male students of medical colleges in the eastern region of Saudi Arabia

**DOI:** 10.4103/0256-4947.72276

**Published:** 2010

**Authors:** Sourabh Aggarwal, Vivek Kesar, Khushbir Singh Bath, Gurpreet Multani

**Affiliations:** a From the University College of Medical Sciences, New Delhi, India drsourabh79@gmail.com; b From the Vardhman Mahavir Medical College, New Delhi, India; c From the Dayanand Medical College and Hospital, Ludhiana, India; d From the Government Dental College, Amritsar, India

**To the Editor**: I read the article ‘Water pipe (shisha) smoking among male students of medical colleges in the eastern region of Saudi Arabia.^1^ by Taha et al with interest. I applaud the authors for conducting such study and would like to share my views on the same.

The study reported that significantly higher proportion of students were from the College of Medicine as compared to the other two. However, this statement can be misinterpreted since a quarter of students from the College of Dentistry (29.41%, 15 out of 51) were ‘shisha’ smokers as compared to the other two groups namely, College of Medicine 8.44% (19 out of 225) and College of Applied Medical Sciences (13.68%, 13 out of 95). It would have been interesting to know whether any attempt was done to conceal the identity of the students while questionnaires were filled since it could have made significant impact on the outcome of the study involving such a sensitive issue. Nonetheless, I really appreciate the efforts of authors to conduct such a study.
